# Integrated single-cell and clinical transcriptomic analysis identifies blunted glycolytic activation as a hallmark of maladaptive repair in renal ischemia–reperfusion

**DOI:** 10.1080/0886022X.2025.2549400

**Published:** 2025-08-28

**Authors:** Alexandre Torck, Frédéric Sangla, Hervé Quintard, Maarten Naesens, David Legouis

**Affiliations:** ^a^ Department of Intensive Care Medicine, Fribourg Hospital, Fribourg, Switzerland; ^b^ Department of Microbiology, Immunology and Transplantation, KU Leuven, Leuven, Belgium; ^c^ Division of Intensive Care Medicine, Department of Anesthesiology, Clinical Pharmacology, Critical Care and Emergency Medicine, Geneva University Hospital, Geneva, Switzerland; ^d^ Laboratory of Nephrology, Department of Physiology and Cell Metabolism, University of Geneva, Geneva, Switzerland

**Keywords:** AKI, transplantation, metabolism, glycolysis

## Abstract

Acute kidney injury (AKI) is a common and increases risk of chronic kidney disease (CKD). While mitochondrial dysfunction drives maladaptive repair, the role of glycolysis in renal recovery remains unclear.

Here, we integrated single-cell transcriptomic data from mouse models with clinical transcriptomic and functional data from kidney transplant recipients to investigate how glycolysis influences epithelial repair. Trajectory inference and metabolic flux estimation revealed divergent cellular fates: cell with active glycolysis achieved recovery, while those with blunted glycolysis followed, maladaptive paths.

These results were confirmed in an independent single-cell dataset and validated clinically in reperfusion biopsies. A machine-learning model trained on clinical parameters identified patients with underactive glycolysis at reperfusion; this subgroup showed impaired long-term recovery.

Although causality is unproven, early glycolytic activation appears linked to regenerative repair. Glycolysis thus emerges as both a biomarker of epithelial fate and a potential therapeutic target to limit CKD progression.

## NEW & NOTEWORTHY

Contrary to the view of glycolysis as a stress marker, we show it is a hallmark of successful epithelial repair. Single-cell and clinical data reveal blunted glycolysis as a consistent feature of maladaptive recovery, positioning glycolysis as a critical checkpoint in renal regeneration..

## Introduction

Acute kidney injury (AKI) is a serious and often life-threatening condition, particularly in critically ill patients [1]. It is characterized by a rapid (≤7 days) and potentially reversible (≤3 months) [2] decline in kidney function, leading to an increased risk of morbidity, mortality, and progression to chronic kidney disease (CKD) [1,3–5]. However, the mechanisms governing renal recovery versus maladaptive repair remain incompletely understood.

Recent research has identified metabolic dysfunction, particularly mitochondrial impairment and disrupted fatty acid oxidation (FAO), as key drivers of AKI pathophysiology and CKD progression [6–14]. Concurrently, a metabolic shift toward glycolysis occurs during AKI [15], yet its precise role in determining renal outcomes remains debated. Some studies suggest that enhanced glycolysis supports tubular repair [16–19], while others indicate that its dysregulation may contribute to fibrosis and chronic dysfunction [20–22].

This study aims to elucidate the impact of glycolysis on renal injury and recovery by integrating single-cell transcriptomic analyses and data from kidney allograft recipients. By characterizing metabolic trajectories associated with successful repair or maladaptive fibrosis, we seek to identify potential therapeutic targets for improving kidney recovery and reducing CKD risk.

## Methods

### Single-nucleus RNA sequencing

We obtained publicly available renal single-cell RNA sequencing (scRNA-seq) and single-cell combinatorial indexing RNA-sequencing data [23] from the Gene Expression Omnibus (GEO) repository (accession number GSE139107). To ensure high data quality, we implemented stringent quality control measures, excluding low-quality cells, doublets, and apoptotic cells—defined as those with fewer than 500 or more than 7,000 detected genes, or fewer than 1,000 unique molecular identifiers (UMIs). DoubletFinder was applied to identify and remove multiplets. Data normalization and variance stabilization were performed using the SCTransform function, employing a Gamma-Poisson (glmGamPoi) distribution to correct for overdispersion.

For dataset integration, we utilized reciprocal principal component analysis (rPCA) in Seurat. A set of 3,000 highly variable features was selected to identify integration anchors, and the first 30 principal components were used for neighbor searches, setting the number of neighbors to 20. Clustering was performed using the FindClusters() and FindNeighbors() functions.

To annotate cell types and assign proximal tubule subpopulations, we used the scType algorithm, which performs supervised cell-type classification based on curated gene signatures. We applied scType to the Proximal Tubule (PT) compartment using the gene markers curated by Kirita et al. [23] allowing consistent re-identification of subtypes including S1, S2, S3, injured S1/S2, injured S3, repairing PT, severe injured PT, and failed-repair PT.

To estimate cellular glycolysis flux, we employed the single-cell flux estimation analysis (scFEA) algorithm [24], which enables inference of cell-wise metabolic fluxes from scRNA-seq data. Specifically, we estimated the flux of pyruvate-to-lactate conversion. The activity across pseudotime was modeled using a generalized additive model to capture dynamic metabolic changes.

### Single-cell combinatorial indexing RNA-sequencing

To assess the robustness of our findings, we analyzed an independent single-cell combinatorial indexing RNA-seq (sci-RNA-seq) dataset from Li et al. [25] (GSE190887), in which mice were subjected to unilateral renal ischemia-reperfusion injury and sacrificed at different post-injury time points. To ensure methodological diversity and assess sensitivity to analytical choices, we deliberately applied a workflow distinct from that used for our primary dataset.

We imported raw count data and sample annotations, reconstructed the gene expression matrix, and created a Seurat object. Cells were filtered to retain only proximal tubular cells based on previously annotated subtypes, and control samples were excluded. Cells with ambiguous or missing annotations were also removed.

To account for batch effects, we performed normalization and variable feature selection independently for each library. Batch-corrected datasets were then merged using Seurat’s JoinLayers functionality. Dimensionality reduction was conducted using PCA followed by UMAP and PHATE projections. Clusters were visualized and identified using previously defined epithelial subtypes.

We then used the PHATE embedding as input to Slingshot for pseudotime inference, setting ‘PT-AcInj’ as the root. To quantify glycolytic activity, we used the irGSEA package to compute singscores for the REACTOME_GLYCOLYSIS gene set, using a batch-processing pipeline to ensure computational scalability. Glycolysis scores were added to the Seurat metadata. We then fit smooth curves along pseudotime using tradeSeq, enabling statistical modeling of glycolysis changes along each trajectory.

### Allograft kidney recipients and transcriptomic profiling

A total of 42 kidney allograft recipients were enrolled at the University Hospitals of Leuven. Each patient underwent protocol biopsies at four defined time points: (i) before implantation (after flushing and cold storage), (ii) immediately after reperfusion, and (iii–iv) at 3 and 12 months post-transplantation. Genome-wide gene expression profiling was performed using bulk RNA sequencing, as previously described [26]

### Estimation of glycolytic activity

To quantify glycolytic activity at the tissue level, we applied the singscore algorithm [27] to normalized gene expression data from each biopsy. Gene sets were curated from the REACTOME collection (MSigDB v1.14). To increase pathway specificity, genes shared between glycolysis and gluconeogenesis modules were excluded prior to scoring. This yielded a single-sample enrichment score for each biopsy, reflecting transcriptome-inferred glycolytic activity, which served as the reference for subsequent modeling.

### Modeling expected glycolysis from clinical features

To model the transcriptome-inferred glycolytic activity, we trained a suite of supervised machine-learning models using the caret package (v7.0-1), with input features including donor and recipient characteristics such as age, sex, donor type, cause of death, cold and warm ischemia times, and perioperative parameters. No transcriptomic data were used as predictors.

Eight modeling approaches were evaluated:

- Random Forest (rf)
- Extreme Gradient Boosting (xgbTree)
- Support Vector Machine with polynomial kernel (svmPoly)
- Regularized Random Forest (RRF)
- LASSO regression (glmnet)
- Stepwise linear regression (lmStepAIC)
- Kernel-regularized least squares (krlsPoly)
- Neural networks (neuralnet).

All models were trained on the full dataset using repeated 10-fold cross-validation (5 repetitions), with hyperparameter optimization *via* internal grid search. Predictive performance was assessed *via* out-of-bag R-squared (R^2^) and root mean square error (RMSE). The best-performing model, based on maximal R^2^, was used to generate patient-level predictions of expected glycolytic activity.

These predicted values were then compared to the observed transcriptomic glycolysis scores to compute standardized residuals (observed − predicted). Patients were subsequently stratified into three categories:

- Under-glycolytic: residuals < 15th percentile.
- Normal: residuals between 15th and 85th percentiles.
- Over-glycolytic: residuals > 85th percentile.

This framework allowed us to detect glycolytic outliers—individuals with transcriptomic activity that deviated significantly from their predicted baseline—potentially reflecting biologically or clinically relevant dysregulation.

## Results

**Figure 1.**
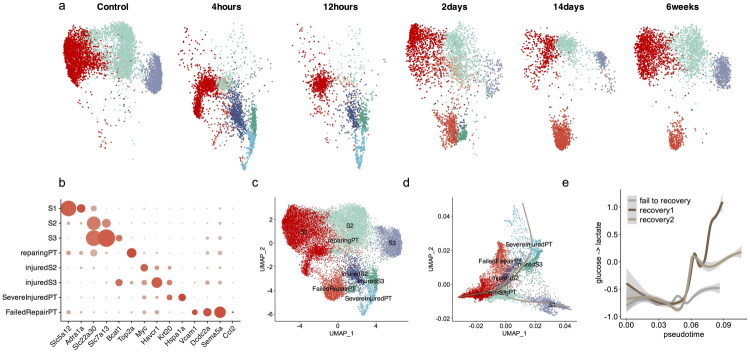
Divergent repair trajectories with different metabolic profile in proximal tubular cells (PT) following ischemia-reperfusion injury. (a) UMAP projection of the integrated snRNA-seq dataset split by time after IRI and colored according to cluster annotation, (b) Dot plot displaying mean marker gene expression and proportion of expression per renal cell type for cluster annotation, (c) UMAP projection of the integrated snRNA-seq dataset with annotated clusters, (d) density UMAP projection with inferred trajectories displaying the normal recovery and fail to recovery lineages and (e) pyruvate to lactate flux estimation along inferred pseudotime and across lineages.

To assess changes in PT cells glycolysis after acute kidney injury (AKI), we analyzed a time-series single-cell transcriptomic dataset following bilateral ischemia-reperfusion injury (IRI) in mice [23]. After data integration and normalization, we subsetted PT cells and recomputed the UMAP projection. PT cells displayed a marked early transcriptomic shift, with no overlap between baseline and 4-h post-injury profiles. Over time, some cells gradually returned to a baseline-like transcriptomic state, while others displayed persistent abnormalities, suggesting maladaptive repair (Figure 1a).

To refine cell state identification, we applied high-resolution clustering followed by transfer learning using scType, leveraging curated marker genes from Kirita et al. This approach allowed us to annotate the original PT subtypes, including S1, S2, S3, injured S1/S2, injured S3, repairing PT, severe injured PT, and failed-repair PT (Figure 1b,c).

We next applied PHATE embedding and Slingshot trajectory inference, which revealed three distinct lineage trajectories originating from the severe injured states: two leading to successful recovery toward S1/S2 and S3 PT, and one corresponding to failed repair (Figure 1d).

We then compared the estimated pyruvate-to-lactate flux along these three trajectories using scFEA. Glycolytic activity was initially low across all lineages but rose progressively along the regenerative paths, reaching sustained high levels. In contrast, cells on the failed-repair trajectory exhibited persistently low glycolytic activity throughout pseudotime. (Figure 1e). These findings suggest that glycolysis may be required to support normal PT repair.

**Figure 2.**
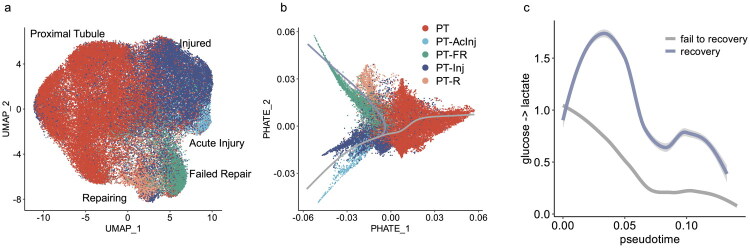
Validation of glycolytic trajectory patterns in an independent dataset. (a) UMAP projection of the integrated sci-RNA-seq dataset from Li et al., with proximal tubule clusters annotated according to the original study. (b) PHATE embedding illustrating two inferred trajectories emerging from the injured state: one leading to normal recovery and the other to failed repair. (c) Estimated pyruvate-to-lactate flux along pseudotime for each trajectory, showing sustained glycolytic activation in the recovery lineage and a decline in the failed-repair branch. PT Proximal Tubule; PT-AcInj Proximal Tubule Acute Injury; PT-FR Proximal Tubule Failed Repair; PT-Inj Proximal Tubule Injured; PT-R Proximal Tubule Repairing.

To validate these findings, we analyzed an independent dataset from Li et al.  [25], which applied single-cell combinatorial indexing RNA-sequencing at multiple time points after unilateral IRI to profile over 200,000 mouse kidney cells. We subset proximal tubule cells, performed data normalization and integration, and annotated the resulting clusters using labels from the original study (Figure 2a).

Despite differences in injury model, sequencing platform, and analytical pipeline, the epithelial trajectories identified in this dataset closely mirrored those from our primary analysis, again revealing distinct paths for recovery and failed repair (Figure 2b). Notably, glycolytic activity was again selectively upregulated along the regenerative lineage and declined along the maladaptive trajectory (Figure 2c**).**

To extend our findings to a clinically relevant context, we analyzed bulk RNA sequencing data from renal allograft biopsies collected immediately after reperfusion—a condition that closely mirrors the ischemia-reperfusion setting of our murine model. Glycolytic activity was quantified in each biopsy using the Singscore algorithm, referencing curated gene sets from the REACTOME database.

**Figure 3.**
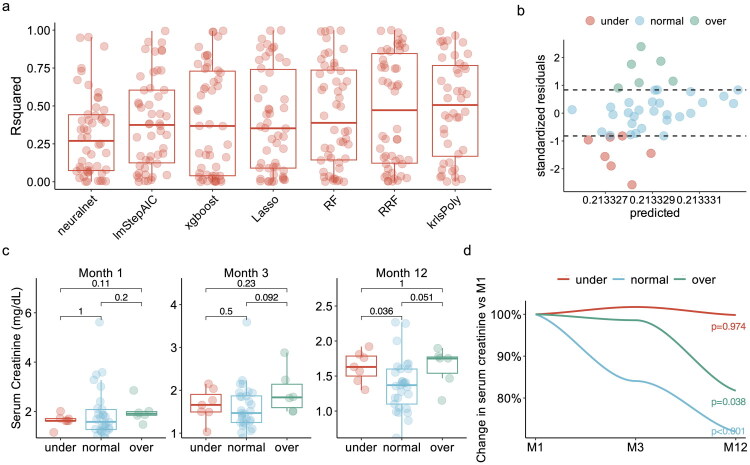
Glycolytic underactivation at reperfusion predicts impaired kidney recovery after transplantation. (a) Performance of machine-learning models in predicting transcriptome-inferred glycolytic activity at the time of reperfusion, assessed using out-of-bag R-squared (R^2^) values from repeated cross-validation. (b) Distribution of standardized residuals (observed minus predicted glycolysis), used to classify patients into under-glycolytic (<15th percentile), normal (15th–85th percentile), or over-glycolytic (>85th percentile) groups. (c) Serum creatinine levels measured at 1, 3, and 12 months post-transplantation across the three glycolytic response groups. (d) Longitudinal changes in serum creatinine relative to Month 1, stratified by glycolytic group. Temporal trends were modeled using LOESS smoothing. Neuralnet – Neural network-based regression modeldlmStepAIC – Linear regression model with stepwise selection using Akaike Information Criterion (AIC)dxgboost – Extreme Gradient Boosting, a tree-based ensemble learning methoddLasso – Least Absolute Shrinkage and Selection Operator, a regression method for feature selection and regularizationdRF – Random Forest, an ensemble learning method using multiple decision treesdRRF – Regularized Random Forest, a variation of Random Forest with feature selectiondkrlsPoly – Kernel Regularized Least Squares with polynomial kernel.

To identify patients exhibiting abnormal glycolytic responses, we first established a population-level baseline of expected glycolysis using only donor and recipient clinical parameters. Multiple machine-learning models were trained to predict transcriptome-inferred glycolytic activity at the time of reperfusion, based exclusively on these non-transcriptomic features. Among the eight models tested, the Kernel Regularized Least Squares model with a polynomial kernel (krlsPoly) demonstrated the highest predictive performance and was selected for subsequent analysis (Figure 3a). For each individual, we then computed standardized residuals (observed minus predicted glycolysis) to quantify deviation from the expected glycolytic activity. Based on these residuals, patients were stratified into three categories: under-glycolytic (<15th percentile), normal (15th–85th percentile), and over-glycolytic (>85th percentile) (Figure 3b).

Serum creatinine was then tracked longitudinally at 1, 3, and 12 months post-transplantation, revealing distinct recovery patterns across glycolytic subgroups (Figure 3c–d). While baseline creatinine levels at one month were comparable between the under- and normal-glycolytic groups (median 1.6 vs. 1.6 mg/dL, *p* = 1.0), the under-glycolytic group showed significantly less improvement at one year (median 1.6 vs. 1.4 mg/dL, *p* = 0.04). In contrast, both normal and over-glycolytic patients exhibited progressive reductions in creatinine over time, consistent with better renal recovery.

These observations reinforce the importance of glycolytic activation during the early reperfusion phase and suggest that failure to mount a timely metabolic response may impair subsequent renal repair—even in the clinical setting of transplantation.

## Discussion

Our findings suggest a beneficial role of glycolysis in the renal response to injury. A strong and rapid glycolytic response is associated with successful repair of PT cells and favorable kidney function recovery. Conversely, impaired glycolysis correlates with maladaptive repair and poor renal recovery.

The kidney has the second highest mitochondrial density after the heart, reflecting its high metabolic demand to actively pump the electrochemical gradients required for tubular reabsorption [28,29]. Under physiological conditions, ATP production relies primarily on oxidative phosphorylation, fueled by FAO, with low glycolytic activity [30,31]. However, under injury, kidneys have been shown to undergo a metabolic switch toward glycolysis [15].

High glycolytic flux has been implicated in multiple regenerative contexts. It promotes self-renewal by reprogramming somatic cells into induced pluripotent stem cells [32,33], supports proliferation and biomass synthesis [34,35], sustains redox balance *via* the pentose phosphate pathway [36,37], and provides oxygen-independent energy during ischemia [38]. By supporting these critical functions, injury-induced glycolysis may serve as a key metabolic determinant of epithelial repair, particularly during the acute phase.

Across models, the regenerative trajectory was consistently marked by selective glycolytic upregulation. In parallel, renal allograft recipients with blunted glycolytic activation at reperfusion exhibited worse functional outcomes at one year. These findings suggest a form of metabolic reprogramming, in which glycolysis may be actively engaged to support the energy and biosynthetic requirements of epithelial repair. This supports the emerging concept that glycolytic activation, though often viewed as a marker of mitochondrial dysfunction, can also reflect an adaptive, pro-regenerative response in injured proximal tubule cells, depending on timing, severity, and cellular context [35,39].

Importantly, our conclusions rely on transcriptomic data and trajectory inference, both of which have inherent limitations. Pseudotime provides a qualitative, not absolute, ordering of cellular states and can be influenced by data sparsity, clustering choices, and dimensionality reduction. The shape of the inferred trajectories should not be overinterpreted, as they vary across models and do not reflect synchronized real-time dynamics. Rather than focusing on the morphology of the curves, our analysis emphasizes the consistent association between glycolytic induction and the regenerative lineage. This pattern was independently observed in a distinct single-cell dataset using a different sequencing platform and an alternative analysis pipeline, reinforcing the robustness of our findings.

Another key limitation is the unresolved question of causality, whether glycolysis actively promotes repair or merely reflects a more favorable cellular state, while cells with blunted glycolytic responses may simply be too damaged to recover. The timing and reversibility of metabolic reprogramming are also likely to be critical. Our findings underscore the need for functional studies to determine whether impaired glycolysis is a driver or consequence of failed regeneration.

To address this, we propose that future studies employ inducible, tubule-specific genetic models to modulate key glycolytic enzymes and evaluate the functional consequences of altering glycolytic flux during the repair phase. Crucially, such interventions should be temporally controlled to assess whether early and reversible glycolytic activation enhances recovery. When combined with pharmacologic strategies (e.g., 2-deoxyglucose or dichloroacetate), these tools may help clarify whether glycolytic modulation is beneficial or detrimental, depending on the timing of intervention, the severity of injury, and the specific cellular context.

In summary, while our study does not demonstrate causality, it reveals reproducible glycolysis upregulation linked to regenerative versus maladaptive outcomes across multiple experimental systems. By leveraging single-cell resolution and integrating transcriptomic profiling with clinical outlier detection based on predicted glycolytic activity, our work lays the foundation for future mechanistic investigations into the metabolic checkpoints that govern epithelial regeneration. A better understanding of these processes could inform new therapeutic strategies to promote kidney repair and mitigate progression to CKD.
